# Geriatric rehabilitation in older patients with cardiovascular disease: a feasibility study

**DOI:** 10.1007/s41999-018-0119-2

**Published:** 2018-10-12

**Authors:** Eléonore F. van Dam van Isselt, Jan van Wijngaarden, Dirk J. A. Lok, Wilco P. Achterberg

**Affiliations:** 1 Department of Public Health and Primary Care, Leiden University Medical Centre, PO Box 9600, 2300 RC Leiden, The Netherlands; 2 Zorggroep Solis, Deventer, The Netherlands; 3 Department of Cardiology, Deventer Hospital, Deventer, The Netherlands

**Keywords:** Geriatric rehabilitation, Cardiovascular disease, Feasibility, Heart failure, Cardiac surgery

## Abstract

**Purpose:**

Cardiac rehabilitation in older patients after hospitalization because of cardiovascular disease is recommended. However, many older patients do not receive cardiac rehabilitation in daily practice, due to lack of referral and poor adherence. This can be related to impaired clinical and functional status of these patients, who are more likely to present with frailty, frequent comorbidities, and disability. Geriatric rehabilitation might be a possible solution to reduce barriers to cardiac rehabilitation attendance. We developed and implemented an inpatient geriatric rehabilitation programme that was provided immediately after discharge from the hospital, for older patients with a significant functional decline during hospital admission because of cardiovascular disease: ‘the GR-cardio programme’. The primary aim of the present study is to investigate feasibility of the GR-cardio programme.

**Methods:**

This is a retrospective real-life feasibility study describing a consecutive series of older patients receiving the GR-cardio programme, with no control group. All patients had been hospitalized because of cardiovascular disease. Data on patient characteristics, functional status, health-related quality of life (HRQoL), readmissions, and mortality were collected from the patients file on admission, at discharge and 6 months after discharge from the GR-cardio programme. Feasibility of the programme was evaluated using the following outcomes: recruitment, resulting sample characteristics, safety, and preliminary evaluation of patients’ responses to the GR-cardio programme.

**Results:**

In total, 58 patients [mean age 78.8 (± 9.8) years; 43% male] were included in the study. On admission, functional status and HRQoL were severely impaired but showed clinically relevant improvements. During the programme, three patients died. Eighty-three percent of all patients were discharged back home after completing the rehabilitation programme with a mean length of 38 days. Mortality rate during follow-up was the highest in patients with heart failure (32%).

**Conclusions:**

This study indicates that geriatric rehabilitation for patients with cardiovascular disease is feasible. Furthermore, our results show that the GR-cardio programme can probably offer substantial benefits for patients in terms of improving functional status and HRQoL.

## Background

Cardiovascular disease represents a major burden for individual patients and societies in terms of health care costs and organization. Because cardiovascular disease is strongly related to age and in view of the worldwide aging populations, the number of older patients with cardiovascular disease is increasing rapidly [1]. This trend raises a particular challenge for cardiac rehabilitation of older patients, hospitalized for cardiovascular disease.

In older patients, cardiovascular disease is often accompanied by other age-related problems that could interfere with rehabilitation. Complex multi-morbidity, polypharmacy, cognitive problems, and impaired functional, nutritional, and psychosocial status are prevalent in older adults. Nevertheless, research has shown that cardiac rehabilitation can offer substantial benefits for older patients with heart failure [2–4], after coronary artery bypass graft (CABG) surgery and/or heart valve replacement (HVR) [5, 6], and after an acute cardiac event (unstable angina pectoris (AP), acute myocardial infarction (AMI)) [7]. Moreover, older patients with cardiovascular disease are, perhaps, most likely to benefit from the multidisciplinary approach that characterises cardiac rehabilitation [2]. However, despite proven benefits, many older patients with cardiovascular disease do not receive cardiac rehabilitation in daily practice [8–12]. Several reasons for this underuse can be identified. First, many physicians do not recommend cardiac rehabilitation to their older patients. This lack of referral can be related to the more impaired clinical and functional status of these patients, who are more likely to present with frailty, multi-comorbidities, and disability [12]. Second, patients that are referred are less likely to participate, also due to poor health status caused by comorbidities and other age-related problems [3]. Finally, hospitalized older patients often experience a (temporarily) care dependence. This requires a specific setting for rehabilitation including care and training of activities of daily living (ADL) and a fluent transition from hospital admission to rehabilitation setting.

Geriatric rehabilitation might be a possible solution to reduce barriers to cardiac rehabilitation attendance. Geriatric rehabilitation is defined as ‘evaluative, diagnostic, and therapeutic interventions whose purpose is to restore functional ability or enhance residual functional capability in older adults with disabling impairments’ [13]. In the Netherlands, geriatric rehabilitation is provided as a form of post-acute rehabilitation, offered at skilled nursing facilities (SNF), usually situated in nursing homes, and organized as structured care pathways in close collaboration with several medical departments of adjacent hospital(s). Geriatric rehabilitation has proven to have the potential to restore or improve functional independence, activity, and social participation, and decrease mortality and nursing homes admissions in geriatric patients after acute hospital stay [14]. However, data on geriatric rehabilitation programmes specifically designed for older patients with cardiovascular disease are lacking, indicating the need for research to provide data on development, implementation, and outcomes of specific cardiovascular geriatric rehabilitation programmes [14].

In 2016, we developed and implemented an inpatient post-acute geriatric rehabilitation programme for older patients with a significant functional decline during hospital admission because of cardiovascular disease: ‘the GR-cardio programme’. Aim of the programme is to restore functional capacity to such an extent that discharge back home is possible, improve quality of life and self-management, and prevent hospital readmissions. When the programme was first developed, it was primarily aimed at patients hospitalized because of acute-on-chronic heart failure. However, during implementation and with time, clinical practice showed that almost half of the patients admitted for the GR-cardio programme were patients after CABG surgery and/or HVR. A few patients were indicated for the GR-cardio programme after an acute cardiac event (AP and AMI). This development in clinical practice has raised the question if separation into two subgroups of patients, HF and non-HF, might be useful. Therefore, the primary aim of the present study is to investigate feasibility of the GR-cardio programme. Secondary aim is to investigate if differentiating the GR-cardio programme into two separate sub-programmes could be useful, based on different patient characteristics, problems, and needs.

## Methods

### Design

**Fig. 1:**
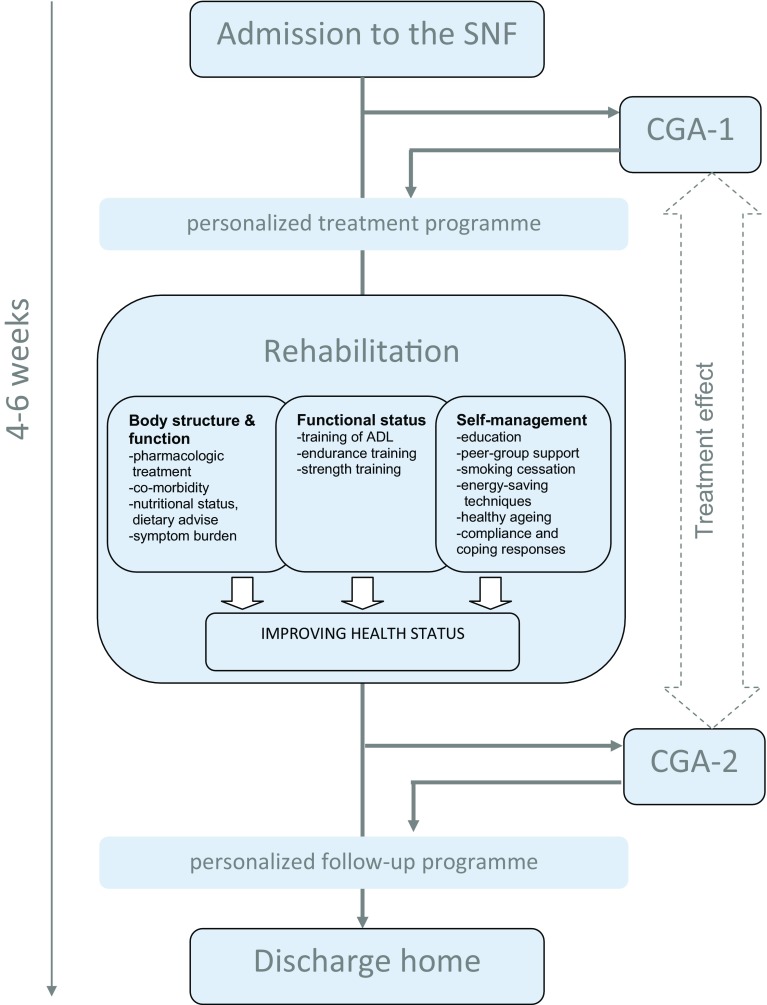
Flowchart of the GR-cardio programme.* GR* geriatric rehabilitation,* SNF* skilled nursing facility,* CGA* comprehensive geriatric assessment,* ADL* activities of daily living

This is a retrospective real-life feasibility study describing a consecutive series of patients, all receiving the GR-cardio programme, with no control group. All patients that followed the GR-cardio programme between May 2016 and September 2017 were included in this study. We collected data from the medical charts on patient characteristics and outcome measurements at three time points: (1) admission to the GR-cardio programme; (2) discharge from the GR-cardio programme; (3) 6 months after discharge from the GR-cardio programme (Fig. 1). As this was a retrospective medical chart review study that aimed to evaluate the provided care (quality assessment) and data were retrieved from the patients file by the patients physician only and put into an anonymous database, no written informed consent was required.

### Patients and setting

Patients admitted to the hospital because of cardiovascular disease, who were not able to be discharged back home because of high symptom burden and severely limited functional status, were eligible for the GR-cardio programme. Indication for rehabilitation was set by a multidisciplinary team including the patients’ cardiologist, physiotherapist, a specialist cardiac nurse, and the elderly care physician of the geriatric rehabilitation ward. Criteria for rehabilitation were not standardized, but tailored to the individual patients’ needs and problems, depending on their medical, social, and general health status. Furthermore, patients had to be motivated and a multidisciplinary programme had to be deemed necessary to achieve improvement (not only physiotherapy). We defined two subgroups of patients admitted to the GR-cardio programme: HF [patients hospitalized with primary diagnosis of (acute-on) chronic heart failure] and non-HF [patients hospitalized because of CABG surgery and/or HVR or after an acute cardiac event (percutaneous coronary intervention (PCI) or AMI)].

### The GR-cardio programme

In the Netherlands, geriatric rehabilitation is provided at skilled nursing facilities (SNF), usually situated at nursing homes, and consists of a multidisciplinary, patient-centred, restorative inpatient treatment within a therapeutic living environment [15]. The multidisciplinary geriatric rehabilitation team consists of an elderly care physician, skilled nurse, physiotherapist, psychologist, occupational therapist, dietician, speech therapist, and a social worker. Geriatric rehabilitation in the Netherlands is coordinated by the elderly care physician, who is specialized in rehabilitation of older patients with complex health issues and multiple comorbidities [16]. The GR-cardio programme consists of several standard modules addressing different aspects of rehabilitation within three domains: body structure and function, functional status, and self-management (Fig. 1). Patient-centred goal setting and duration of the programme is individually tailored to the patients’ needs and preferences, also based on a standard comprehensive geriatric assessment (CGA) that is assessed within the first 3 days after admission. The modular programme is evaluated weekly and adjusted (as needed) by the multidisciplinary team, in consultation with the patient and his or her relatives. All patients follow a standardized day programme and assignment to therapies is stringent. Standard modules are: physiotherapy (daily, 40 min), occupational therapy (30 min, twice a week), diet advice (30 min, weekly), patient education, peer group support and improvement of self-management through weekly group sessions (60 min), individual coaching by the skilled nurse (daily), and, if indicated, consultation by the psychologist or social worker.

### Measurements and outcome

We collected the following patient and disease characteristics from the patient’s file: age, sex, primary cardiac diagnosis, and number of comorbidities listed [diabetes mellitus (DM), cerebral vascular accident (CVA), chronic obstructive pulmonary disease (COPD), pulmonary hypertension (PHT), hypertension (HT), atrial fibrillation (AF) and renal failure (RF)]. Nutritional status was measured by calculating the body mass index (BMI kg/m^2^) which was divided into four categories, based on international guidelines: underweighted (< 18.5 kg/m^2^), normal weight (18.5–24.9 kg/m^2^), pre-obesity (25–29.9 kg/m^2^), and obesity (> 30 kg/m^2^) [17].

The following measurements were obtained from the CGA on admission to and discharge from the GR-cardio programme. Functional status was measured using the Barthel Index (BI) for activities of daily living (ADL) and the six-minute walking test (6MWT) for exercise capacity. The BI is a widely used, valid, and reliable instrument to assess functional improvement during rehabilitation [18, 19]. Total score ranges from 0 to 20, with 20 representing complete functional independence. The minimal clinically important difference (MCID) of the BI is + 1.85 [20]. A score of < 15 indicates moderate-to-severe ADL impairment. The 6MWT is a commonly used outcome measurement in cardiac rehabilitation and has shown to be responsive to clinical change following rehabilitation [21]. The MCID for the 6MWT for older cardiovascular patients was set at + 54 m [22].

Psychosocial status was measured with the hospital anxiety and depression score (HADS). The HADS is a valid and reliable screening instrument to assess the symptoms of anxiety and depression [23]. It is a 14 items’ scale with two subdomains (anxiety and depression) with seven items which are scored on a Likert scale ranging from zero to three. A score of eight points or higher on either subscale indicates a higher risk for the presence of clinically relevant anxiety or depression.

Health-related quality of life (HRQoL) was measured using the Minnesota living with heart failure questionnaire (MLHFQ), a valid and reliable tool to assess HRQoL in patients with cardiovascular disease and sensitive to change in relation to treatment [24, 25]. Interpretation of scores is not standardized, but a total score < 24 is considered as good HRQoL, from 24 to 45 as moderate impaired and > 45 as severely impaired HRQoL. Improvement on the MLHFQ of 12, 14, or 21 points is considered as slight, moderate and large improvement respectively [26].

Length of admission to the GR-cardio programme (LOA) and cardiovascular-related hospital readmissions and deaths occurred during 6-month post-intervention following the index hospitalization, which were collected from the patients file.

To investigate feasibility of the programme, we focussed on the following objectives and guiding questions, partly adapted from Orsmond et al. [27]: (1) Evaluation of recruitment and resulting sample characteristics; “Can we recruit the appropriate participants?” (2) Evaluation of acceptability, safety and adverse events; “What is the adherence rate to the GR-cardio programme, what is the level of safety and are there any unexpected adverse events?” (3) Preliminary evaluation of patients’ responses to the GR-cardio programme; “Does the programme show promise of being successful within this population?”

### Statistical analysis

All data were processed using SPSS (IBM SPSS Statistics for Windows version 23.0). Descriptive analyses were used for general patient and disease characteristics and data from measurements on admission. Categorical variables are described as frequencies, while continuous variables are tested for normality and presented as mean and SD. Comparisons between groups (HF and non-HF) on admission to the programme were performed using independent samples *t* tests and Chi-square tests, as appropriate. Changes within groups of three outcome measurements (BI, 6MWT, and HRQoL) during the GR-cardio programme (scores at discharge compared to scores on admission) were tested using paired samples *t* tests. We defined statistical significance at *p* < 0.05 (two-sided level of significance).

## Results

### General characteristics

In total, 58 consecutive patients (25 male) with a mean age of 78.8(± 9.8) years followed the GR-cardio programme within the given period and were included in the study. Table 1 presents patient and disease characteristics of the total group and stratified by primary reason for hospital admission (HF versus non-HF). Of the 31 patients with an index hospitalization for acute-on-chronic HF, 25 patients were diagnosed with HF with reduced ejection fraction (HFrEF) and 6 with HF with preserved ejection fraction (HFpEF). Of the patients in the non-HF group (*n* = 27), 23 were admitted to the GR-cardio programme after they had been hospitalized for CABG and/or HVR surgery and four patients after AMI or PCI.

**Table 1:** Characteristics of the study population on admission to the GR-cardio programme, stratified between the two groups (HF and non-HF)

	Total (*N* = 58)	HF (*N* = 31)	Non-HF (*N* = 27)	*p*
SEX, male, *n* (%)	25 (43)	12 (39)	13 (48)	0.60
AGE, years, mean (SD)	78.8 (9.8)	82.4 (8.9)	74.4 (9.3)	0.002
Comorbidity, *n* (%)				
DM	23 (40)	12 (39)	11 (41)	0.9
TIA/CVA	12 (21)	11 (35)	1 (4)	0.003
COPD	19 (33)	14 (45)	5 (18)	0.05
PHT	4 (7)	4 (13)	0 (0)	0.11
HT	24 (41)	14 (45)	10 (37)	0.60
AF	25 (43)	13 (42)	12 (44)	0.99
RF	14 (24)	11 (35)	3 (11)	0.04
Total comorbidity score, mean (SD)	2.3 (1.4)	2.5 (1.4)	2.0 (1.4)	0.17
BMI, kg/m^2^, mean (SD)	26.0 (4.6)	26.1 (5.3)	26.0 (3.6)	0.95
BMI_categories, *n* (%)				
Underweight	0 (0)	0 (0)	0 (0)	0.49
Normal	27 (46.5)	15 (48.4)	12 (44.5)	
Pre-obesity	24 (41.4)	11 (35.5)	13 (48.1)	
Obesity	7 (12.1)	5 (16.1)	2 (7.4)	
BI, mean (SD)	14.9 (3.4)	14.5 (3.7)	15.3 (3.1)	0.35
6MWT, m, mean (SD)	122 (96)	104 (86)	142 (103)	0.14
HADS-A, mean (SD)	5.4 (4.2)	4.1 (3.1)	7.0 (5.1)	0.03
HADS-D, mean (SD)	6.0 (5.0)	4.3 (4.1)	8.2 (5.6)	0.01
MLHFQ, mean (SD)	56.5 (20.0)	53.3 (14.3)	60.4 (25.0)	0.21

*HF* heart failure group, *non-HF* non-heart heart failure group, *DM* diabetes mellitus, *CVA* cerebral vascular accident, *COPD* chronic obstructive pulmonary disease, PHT pulmonary hypertension, *HT* hypertension, *AF* atrial fibrillation, *RF* renal failure, *BMI* body mass index, *BI* Barthel index, *6MWT* six-minute walking test, *HADS* hospital anxiety (A) and depression (D) score, *MLHFQ* Minnesota living with heart failure questionnaire

Comorbidities were frequent. Two-third (*n* = 39) of the patients suffered from two or more of the listed comorbidities. No difference was found in total amount of comorbidities between the HF and non-HF groups. However, CVA/TIA (35 vs 4%; *p* = 0.003), COPD (45 vs 18%; *p* = 0.05), and renal failure (35 vs 11%; *p* = 0.04) were more prevalent in the HF group compared to the non-HF group.

Mean BMI was > 25 kg/m^2^, indicating that (pre)-obesity was frequent in these patients. In total, more than half of the patients (*n* = 31) were (pre)-obese. Functional status was poor in both groups, but showed a trend towards even lower scores in patients with HF, but differences were not significant. ADL status on admission showed moderate care dependence. Although mean scores on the HADS in the total group were below the threshold, patients in the non-HF group had a moderate-to-severe risk for the presence of an anxiety disorder or depression.

### Changes in functional status and HRQoL during the GR-cardio programme

Table 2 presents changes in functional status and HRQoL during the GR-cardio programme. On admission to the programme, functional status and HRQoL were severely impaired; patients were ADL-dependent, and exercise capacity was poor, especially in patients with HF. During the programme, functional status and HRQoL improved significantly and to a clinically relevant extent. During the GR-cardio programme, ten patients were readmitted to the hospital, of whom three did not return to the SNF; two were discharged home from the hospital; one patient died in the hospital due to a non-cardiac cause. Three other patients (two HF and one non-HF) died at the SNF: one patient with HF died unexpectedly due to an acute cardiac event and in two other patients rehabilitation was not feasible and palliative care was given. Overall, 48 patients (83%) were discharged back home, five patients went to a residential care facility, and one patient was admitted to a nursing home. None of the patients dropped out from the programme. Mean LOA to the SNF was 38.0 (± 19.2) days for the total group with no differences between the two subgroups.

**Table 2:** Changes in functional status and health-related quality of life between admission to and discharge from the GR-cardio programme

	*N*	Admission	Discharge	*p*
Total group				
BI	52	15.1 (3.2)	17.8 (2.0)	< 0.001
6MWT	51	123 (95)	208 (99)	< 0.001
MLHFQ	48	56.6 (20.2)	35.5 (24.4)	< 0.001
HF				
BI	27	14.7 (3.2)	17.2 (2.1)	< 0.001
6MWT	26	132 (88)	192 (82)	< 0.001
MLHFQ	22	54.0 (14.5)	39.6 (22.4)	0.001
Non-HF				
BI	25	15.3 (3.2)	18.5 (1.8)	< 0.001
6MWT	25	113 (90)	223 (96)	< 0.001
MLHFQ	20	59.8 (25.4)	30.6 (20.3)	< 0.001

All values are presented as means (SD)

Abbreviations: HF: heart failure group, non-HF: non-heart failure group, BI: Barthel index, 6MWT: six-minute walking test, MLHFQ: Minnesota living with heart failure questionnaire

### Follow-up after discharge from the GR-cardio programme

During the follow-up period of this study (6 months after discharge from the GR-cardio programme), nine patients were readmitted to the hospital, leading to 13 hospital admissions in total, compared to 42 hospital admissions in the 6 months prior to the hospital admission that preceded admission to the programme. No differences were found in readmission rates between the two groups. During follow-up, nine patients died. Total mortality rate was highest in the HF group: 10 of the 31 patients (32%) died within the time-frame of this study, compared to 3 (11%) in the non-HF group.

## Discussion

### Main findings

The primary aim of this study was to investigate feasibility of the GR-cardio programme. Therefore, we will interpret our results on the basis of the three feasibility guiding questions.

(1) “Can we recruit the appropriate participants?” Our results show that patients admitted to the GR-cardio programme suffered from severely impaired functional status and HRQoL. In addition, patients in the non-HF group experienced clinically relevant symptoms of anxiety and depression. Furthermore, overall mean age was high and comorbidities were frequent. These results confirm the need for specific geriatric rehabilitation programmes for this growing group of patients with cardiovascular disease, complex health issues, and diminished functional status. (2) “What is the adherence rate to the GR-cardio programme, what is the level of safety and are there any unexpected adverse events?” The adherence rate to the programme was high. None of the patients resigned and only three patients were lost after hospital readmission. Considering the high mortality rate during follow-up in the HF group and one death caused by an acute cardiac event during the programme, caution to the feasibility of the programme for older patients with HF is advised. (3) “Does the programme show promise of being successful within this population?” Functional status and HRQoL showed significant and clinically relevant improvement during the programme and 48 of all 58 patients were discharged back home after a mean length of stay of 38 days. These results indicate that the GR-cardio programme is feasible and can probably offer substantial improvements in functional status and HRQoL. Secondary aim was to investigate if differentiating the GR-cardio programme into two separate sub-programmes could be indicated, based on different patient characteristics, problems, and needs. Our results show some significant and clinically relevant differences between the two groups. Patients in the non-HF group were younger and showed higher scores on the HADS, indicating more severe symptoms of anxiety and depression, compared to patients in the HF group.

### Interpretation of findings and relation to literature

Our data are in line with a growing body of evidence, showing that cardiac rehabilitation for very old adults with cardiovascular disease is feasible and effective [2–7]. However, these studies all investigated the other types of cardiac rehabilitation programmes (usually in an outpatient hospital-based setting) and included individuals that showed different patient and disease characteristics, compared to our study. Baldasseroni et al. conducted an observational study in 160 patients aged 75 years and older after an acute coronary event treated with PCI, CABG, and/or HVR, who enrolled an outpatient cardiac rehabilitation programme of 4 weeks [7]. Although age and comorbidity were similar to our patients, disabilities in ADL were considered as exclusion criteria and the mean 6MWT on admission was much higher [397.7(± 93.3) m], compared to our study population. The authors reported a clinically relevant improvement in all indexes of physical improvement (including the 6MWT). Furthermore, the authors concluded that older adults with the greatest physical impairment are probably the most appropriate candidates for cardiac rehabilitation that incorporates physical exercise training from which they seem to benefit the most [7]. Similar results were obtained in a study on the effects of a cardiac rehabilitation programme after cardiac surgery that lasted for 20 days [6]. In this randomized-controlled trial, mean baseline values of the 6MWT were, however, again much higher [mean 6MWT 296 (± 84) m], compared to our results, and no information on ADL status of the participants was reported. In studies reporting on the effect of cardiac rehabilitation in patients with HF, mean baseline values of the 6MWT were also higher [12], or quality of life was less limited and patients were younger [2]. Thus, although we could not find studies that included patients with similar characteristics (age and comorbidity), functional status (limited ADL status and poor exercise capacity), and HRQoL compared to our population, literature is in line with our results, indicating that rehabilitation of older patients with cardiovascular disease is feasible and can probably offer substantial benefits. The referenced studies also emphasize the need for development and scientific evaluation of cardiac rehabilitation programmes, especially designed for older patients with severely impaired health status. These patients need a specific setting and programme that is adjusted to their needs and problems. This is in line with the results from the multicentre real-life survey study of Giallauria et al. that provided an insight in the clinical characteristics and course of a very old population receiving cardiac rehabilitation [12]. The authors reported more acute phase complications and comorbidities in older patients, and these patients were also more likely to be discharged to nursing homes and had a higher death rate during cardiac rehabilitation.

Of special interest is our finding that patients in the non-HF group are at risk for an anxiety disorder or depression. Depression and anxiety are known to have a negative effect on outcomes and prognosis in patients after CABG [28, 29]. Cardiac rehabilitation can be effective in reducing anxiety and depression in patients undergoing CABG [30]. These results were, however, derived from studies in younger patients with less comorbidities and better functional status. One study in older patients [mean age 80.3 (± 6.2) years] after transcatheter aortic-valve implementation (TAVI) with similar amount of comorbidities also measured psychological status using the HADS. Mean scores were, however, much lower [HADS_A: 4.4(± 3.3); HADS_D: 5.0(± 3.8)] compared to our subjects [5, 30]. In our clinical practice, this finding has led to an adjustment to the programme as treatment by the psychologist is now considered as standard care in these patients.

### Strength and limitations

To the best of our knowledge, this is the first study to focus on feasibility of a geriatric rehabilitation programme for older patients hospitalized for cardiovascular disease. Although numbers of included patients are small, especially when the two groups are analysed separately, data from all patients admitted to the GR-programme were analysed and no exclusion criteria were applied. Considering this, our study can be seen as a real-life feasibility study and especially in research concerning (very) old patients, observational real-life data are needed [31]. However, we did not investigate all recommended distinctive features of a feasibility study. First, and most important, we could not report on recruitment rate, as data from patients that were indicated but not motivated for the programme were not collected. Second, due to the selection procedure, which was not based on strict inclusion criteria, population bias could be present and might have influenced our sample characteristics. Lack of control group, although self-evident when considering the design and aims of this study, can also be seen as a limitation, because it is plausible that those that received usual care also experienced a significant improvement, especially among survivors [i.e., the before-after changes come from survivors only (survivorship bias)]. Furthermore, we used the MLHFQ to measure HRQoL and this questionnaire has only been validated in patients with HF. Selection of this particular questionnaire for the study is due to the fact that the programme was originally aimed at patients with HF. Therefore, the outcomes of HRQoL in the non-HF group must be considered with caution. We did not collect data on cognitive function of the included patients, because, although part of the CGA, cognitive function was not assessed in a standard and objective way. Therefore, no reliable data could be collected from the medical files. However, as cognitive function in this specific population and setting is very relevant in terms of outcomes and prognosis, standard and objective assessment of cognitive function is advised [3, 32].

### Conclusion and implications

Geriatric rehabilitation for patients with cardiovascular disease is feasible in terms of recruitment of appropriate participants and adherence, and can probably offer substantial improvement in functional status and HRQoL. Caution to the feasibility of the programme for older patients with heart failure is advised and further research is needed. A pilot study should investigate the outcomes of the programme, identify patient characteristics that can predict which older patients with cardiovascular disease are most likely to benefit, especially in older patients with heart failure, and investigate if conducting a randomized-controlled trial can be recommended.
